# Predicting common cardiovascular and non-cardiovascular outcomes after new-onset atrial fibrillation: an analysis from a UK nationwide primary-secondary care linkage dataset

**DOI:** 10.1093/ehjopen/oeag103

**Published:** 2026-06-09

**Authors:** Hiroyuki Yoshimura, A Floriaan Schmidt, Gregory Y H Lip, Rui Providencia

**Affiliations:** Institute of Health Informatics Research, University College London, 222 Euston Road, London NW1 2DA, UK; Institute of Cardiovascular Science, Faculty of Population Health, University College London, London WC1E 6BT, UK; UCL British Heart Foundation Center of Excellence, London WC1E 6BT, UK; Department of Cardiology, Amsterdam Cardiovascular Sciences, Amsterdam University Medical Centres, University of Amsterdam, Meibergdreef 9, 1105 AZ Amsterdam, Netherlands; Liverpool Centre for Cardiovascular Science at University of Liverpool, Liverpool John Moores University and Liverpool Heart & Chest Hospital, Liverpool L14 3PE, UK; Department of Clinical Medicine, Aalborg University, 9220 Aalborg, Denmark; Department of Cardiology, Lipidology and Internal Medicine with Intensive Coronary Care Unit, Medical University of Bialystok, 15-089 Bialystok, Poland; Institute of Health Informatics Research, University College London, 222 Euston Road, London NW1 2DA, UK; Barts Heart Centre, Barts Health NHS Trust, London EC1A 7BE, UK

**Keywords:** Arrhythmia, Dementia, Prediction, Prognosis, Heart

## Abstract

**Aims:**

Atrial fibrillation (AF) increases the risk of major cardiovascular and neurological complications. Utilization of risk schemes for identifying patients at risk for stroke has improved outcomes for patients with AF.

**Objectives:**

Using linked UK primary and secondary care data, we identified risk predictors and develop risk models for heart failure (HF) hospitalization, myocardial infarction (MI) hospitalization, vascular dementia, sepsis, sudden cardiac death (SCD), and all-cause death in the AF population.

**Methods and results:**

We included 198 995 patients diagnosed with AF between January 1998 and May 2016. We developed multivariable Cox regression models to predict outcome incidence after AF diagnosis, using 70% of the data for derivation and 30% for validation. Model performance was assessed using Harrell’s c-index. Predictor variables were selected based on their availability in routine clinical practice. Among the assessed predictors, excessive alcohol intake, HF, MI, sepsis, and stroke showed the strongest associations with adverse outcomes. The prediction models demonstrated fair to moderate discrimination, with c-index values of 0.70 (95% CI 0.70–0.71) for HF hospitalization, 0.64 (95% CI 0.63–0.65) for MI hospitalization, 0.76 (95% CI 0.75–0.77) for vascular dementia, 0.66 (95% CI 0.65–0.67) for sepsis, 0.65 (95% CI 0.62–0.68) for SCD, and 0.71 (95% CI 0.71–0.71) for all-cause death.

**Conclusion:**

In patients with AF, we developed prediction models that demonstrate fair to good performance. Our models, based on variables easily obtainable in routine clinical care, may facilitate patient risk stratification and guide further diagnostic or preventive interventions.

## Introduction

Atrial fibrillation (AF) is one of the most common cardiovascular conditions. The global prevalence of AF in 2023 was estimated to be 59 million individuals and is expected to increase further.^1,2^

Stroke is one of the most serious complications of AF and guidelines recommend risk stratification tools to improve patient management.^3,4^ The CHA_2_DS_2_-VASc score is widely used to better identify patients at risk of stroke and to guide optimal treatment strategies for stroke prevention.^5^ This score has contributed to more appropriate use of oral anticoagulants (OACs), reducing unnecessary anticoagulation in truly low-risk individuals and thereby lowering the risk of bleeding. Multiple observational studies and registries have demonstrated an increase in anticoagulant prescription rates and a decline in AF-related stroke incidence and mortality following the adoption of this risk stratification scheme.^6,7^

Besides stroke, patients with AF are at increased risk of other major cardiovascular and neurological complications.^4,8^ Several studies have reported that AF increases the incidence of heart failure (HF), myocardial infarction (MI), and vascular dementia.^9–12^ Importantly, coexistence of AF with these conditions is associated with a significantly higher risk of mortality compared to AF or each condition alone.^13–15^ Cohort studies indicate AF is associated with an increased risk of sudden cardiac death (SCD).^16^ Infections are also a frequent problem in the AF population.^17^ Sepsis is the most common cause of death in individuals with infection. The World Health Organization estimates that 11 million sepsis-related deaths occur worldwide, representing 20% of all global deaths^18^ In patients with AF, sepsis is associated with longer duration of hospitalization, higher rate of stroke, HF, MI, and mortality.^19^ Timely diagnosis and initiation of antibiotics can prevent part of sepsis-related fatality.

No risk stratification schemes for conditions beyond stroke are currently widely available and/or used in routine AF care. Therefore, developing risk stratification models for cardiovascular and non-cardiovascular outcomes frequently affecting the AF population is essential to improve long-term prognosis and guide more comprehensive management strategies. Using linked UK electronic health records, we aimed to develop prediction models for the risk of MI hospitalization, HF hospitalization, vascular dementia, sepsis, SCD, and all-cause death in individuals with AF.

## Methods

### Data sources

We utilized nationally linked nationwide electronic health records in the UK, which integrated three sources: Clinical Practice Research Datalink for primary care data, Hospital Episodes Statistics for secondary care data, and mortality records from the Office for National Statistic.

## Features (exposures)

Comorbidities and outcomes were identified using READ codes and International Classification of Diseases, Tenth Revision codes, as defined by the Health Data Research UK Phenotype Library.^20^ Potential predictors were selected based on those frequently reported in AF population.^8^ Socioeconomic status was measured using the 2015 English Indices of Deprivation and divided into quintiles. Since up to 20 diagnosis codes are recorded for each hospitalization in HES, we defined HF and MI hospitalizations as those in which HF or MI was listed as the primary (first-listed) diagnosis.

All codes used are listed in Supplementary material online, *Table S1*.

### Study sample

**Figure 1:**
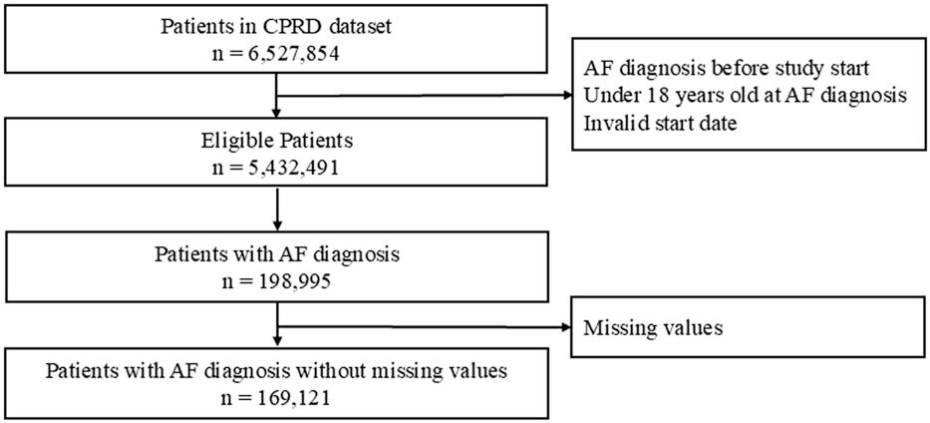
Study flow chart. AF, atrial fibrillation; CPRD, Clinical Practice Research Datalink.

*Figure 1* presents the flowchart of patient selection. From the CPRD dataset, individuals without HES linkage or younger than 18 years were excluded, leaving 6 527 854 patients in the linked datasets between 1 January 1998 and 31 May 2016. We then excluded those with an AF diagnosis recorded before the study start, those aged under 18 years at AF diagnosis, and those with invalid index dates. The final study cohort included 5 432 491 eligible patients, among whom 198 995 received a new AF diagnosis in primary or secondary care during the study period. In total, 29 874 individuals have at least one missing value.

### Statistical methods

The outcomes were HF hospitalization, MI hospitalization, vascular dementia, sepsis, SCD, and all-cause mortality and were selected based on previous work on healthcare utilization and mortality in the AF population.^17^ To develop and validate the prediction model, the dataset was randomly split into a derivation cohort (70%) and a validation cohort (30%). We employed a multivariable Cox regression model in the derivation set to predict the incidence of outcomes following an AF diagnosis, subsequently evaluating its performance in the validation set (i.e. internal validation). To assess the model discrimination in the validation dataset, Harrell’s c-index with 95% confidence intervals (CIs) was calculated. We constructed calibration curves and calculated the intercept and slope with 95% CIs to assess calibration performance by comparing the predicted risk and observed risk. Patients in validation datasets were stratified into quintiles based on predicted risk, with additional categorization of the top 10% and 5% highest-risk groups.

For the prediction models of vascular dementia, patients with a prior history of the vascular dementia were excluded to focus on incident events. In contrast, as recurrent cardiovascular events are common,^21^ for HF and MI hospitalization models, patients with a prior history of HF or MI were not excluded, similar to what has been done for the development of the CHA_2_DS_2_VASc model. The sepsis model also included a prior history of sepsis.

For the main analysis, missing data were assumed to be missing at random and imputed using random forest imputation. In addition, for the sensitivity analysis, we performed complete case analysis and used the Fine–Gray model to account for competing risks when the outcome was other than all-cause death. To assess predictive performance while accounting for competing risks, we calculated time-dependent area under the curves (AUCs) for censored survival data. All statistical analyses were performed using R (version 4.4.1). We followed the Transparent Reporting of a multivariable prognostic model for Individual Prognosis or Diagnosis recommendations.^22^

## Results

Baseline characteristics of the 198 995 patients with new-onset AF diagnosis (mean age: 75.8 ± 12.3 years; 49.1% women; 97.6% White) are presented in *Table 1*. More than half of the patients had hypertension (57.4%). Other common comorbidities included anaemia (18.3%), HF (14.0%), and diabetes (14.0%). A history of stroke or systemic embolism was present in 9.5% of patients, MI in 12.0%, and sepsis in 1.5%.

**Table 1:** Baseline characteristics of the study population

Baseline	
Characteristic	Overall
	*n* = 198 995^a^
Age	75.8 (12.3)
Women	97 803 (49.1%)
Ethnicity	
Non-White	4415 (2.4%)
White	176 530 (97.6%)
Missing	18 050
Most deprived status	45 809 (23.0%)
Smoking	
Non smoker	62 503 (33.8%)
Smoker	122 509 (66.2%)
Missing	13 983
Excessive alcohol intake	5968 (3.0%)
Heart failure	27 762 (14.0%)
Hypertension	114 129 (57.4%)
Diabetes	27 919 (14.0%)
Stroke/SE	18 938 (9.5%)
Myocardial infarction	23 937 (12.0%)
Venous thromboembolism	12 735 (6.4%)
Peripheral artery disease	13 984 (7.0%)
Chronic kidney disease	12 382 (6.2%)
Anaemia	36 432 (18.3%)
Obesity	17 032 (8.6%)
Sepsis	3050 (1.5%)
Vascular dementia	2423 (1.2%)

^a^Mean (SD); *n* (%).

Baseline characteristics of patients with and without missing data are shown in Supplementary material online, *Table S2*. Supplementary material online, *Table S3* presents the number of patients and events for each outcome in the total, derivation, and validation datasets.

### Factors associated with outcome

**Figure 2:**
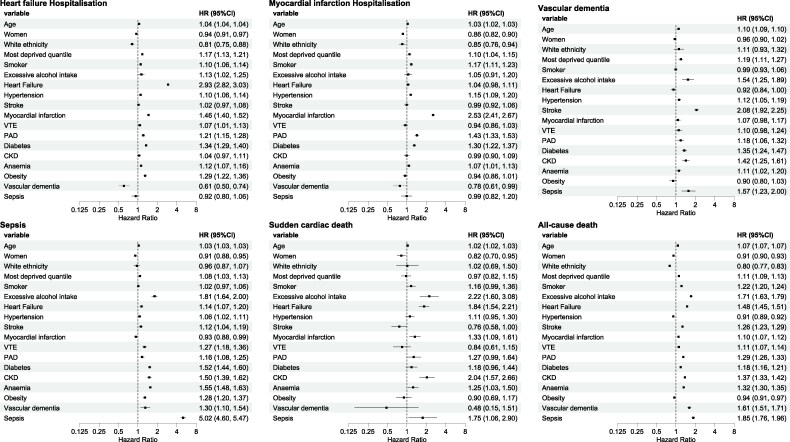
Adjusted hazard ratios for the incidence of each outcome in patients with atrial fibrillation. CKD, chronic kidney disease; PAD, peripheral artery disease; VTE, venous thromboembolism.

*Figure 2* shows the adjusted hazard ratios (HRs) from Cox regression models assessing the incidence of each outcome after AF diagnosis.

Across all six outcomes, older age (HR range: 1.02–1.10) and anaemia (HR range: 1.07–1.55) were associated with higher risk. Most deprived status (HR range: 1.08–1.19), peripheral artery disease (HR range: 1.16–1.43), and diabetes (HR range: 1.18–1.52) were associated with higher risk for outcomes except SCD. Women for all outcomes except vascular dementia (HR range: 0.82–0.94) and white ethnicity for all-cause death, HF hospitalization, and MI hospitalization (HR range: 0.80–0.85) were associated with lower risk. Excessive alcohol intake was associated with higher likelihood of all outcomes except MI hospitalization (HR range: 1.13–2.22). Hypertension was associated with higher risk for HF hospitalization, MI hospitalization, sepsis, and vascular dementia (HR range: 1.06–1.15), but lower risk for all-cause death (HR 0.91, 95% CI 0.89–0.92). Obesity conferred an increased risk of HF hospitalization (HR 1.29, 95% CI 1.22–1.36) and sepsis (HR 1.28, 95% CI 1.20–1.37), while being associated with a reduced risk of all-cause death (HR 0.94, 95% CI 0.91–0.97). Vascular dementia was associated with higher all-cause death (HR 1.61, 95% CI 1.51–1.71) and sepsis (HR 1.30, 95%CI 1.10–1.54), while it was associated with lower HF hospitalization (HR 0.61, 95% CI 0.50–0.74) and MI hospitalization (HR 0.78, 95%CI 0.61–0.99).

The strongest individual associations were observed for previously diagnosed HF with HF hospitalization (HR 2.93, 95% CI 2.82–3.03), and previous MI with MI hospitalization (HR 2.53, 95% CI 2.41–2.67), prior stroke with vascular dementia (HR 2.08, 95% CI 1.92–2.25), prior sepsis with sepsis (HR 5.02, 95% CI 4.60–5.47), excessive alcohol intake with SCD (HR 2.22, 95% CI 1.60–3.08), and sepsis with all-cause death (HR 1.85, 95% CI 1.76–1.96).

### Risk prediction performance

**Figure 3:**
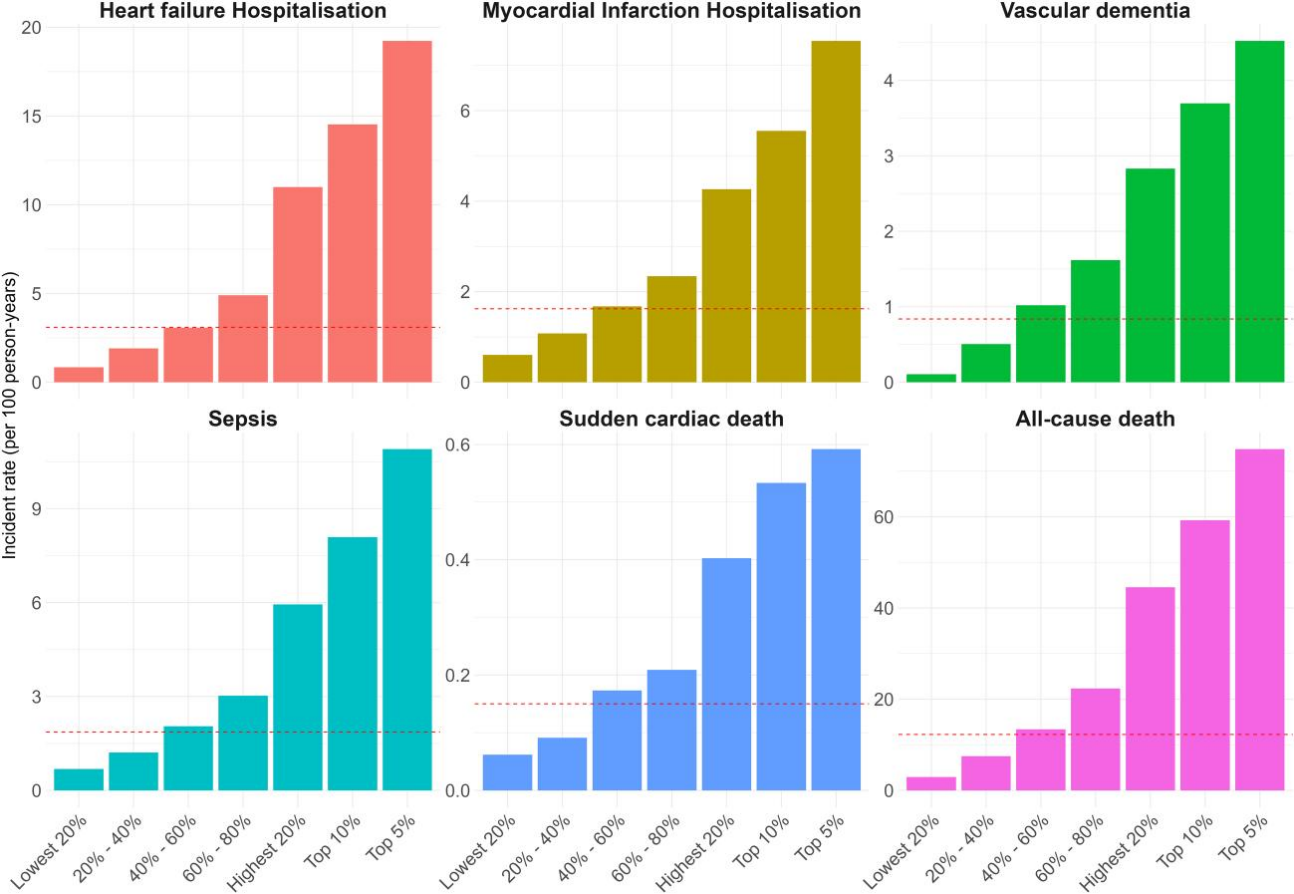
Incidence rates by predicted risk level. Horizontal dashed lines indicate the overall incident rate for each outcome.

When applied to internal validation dataset, the prediction models demonstrated fair to moderate discrimination, with c-index values of 0.70 (95% CI 0.70–0.71) for HF hospitalization, 0.64 (95% CI 0.63–0.65) for MI hospitalization, 0.76 (95% CI 0.75–0.77) for vascular dementia, 0.66 (95% CI 0.65–0.67) for sepsis, 0.65 (95% CI 0.62–0.68) for SCD, and 0.71 (95% CI 0.71–0.71) for all-cause death. *Figure 3* presents incidence rates of the outcomes across risk quintiles and top risk percentiles (top 10% and 5%). Higher risk groups consistently showed higher incidence of the outcome, demonstrating a clear upward trend across strata. Calibration plots indicated good agreement between predicted and observed risk for all outcome (see Supplementary material online, *Figure S1*). The baseline survival estimates at 1, 3, and 5 years are provided in Supplementary material online, *Table S4*.

### Sensitivity analyses

Results from the multivariable models with imputed data were largely in agreement with those obtained from the complete-case analysis. None of the predictors with a 95% CI entirely below one in the primary analysis exceeded one in the imputed models, and vice versa (see Supplementary material online, *Figure S2*). Discriminatory performance remained similar: c-index values were 0.71 (95% CI 0.70–0.71) for HF hospitalization, 0.64 (95% CI 0.63–0.65) for MI hospitalization, 0.78 (95% CI 0.77–0.79) for vascular dementia, 0.66 (95% CI 0.65–0.67) for sepsis, 0.64 (95% CI 0.60–0.67) for SCD, and 0.71 (95% CI 0.71–0.72) for all-cause death. The results from the Fine–Gray model were generally consistent with the main analysis, although some discrepancies were observed. While the direction of effect for certain variables shifted, the overall predictive performance remained stable. The AUC at 1 year after AF diagnosis was comparable to the c-index in the main analysis (HF hospitalization: 0.69, MI hospitalization: 0.63, vascular dementia: 0.74, sepsis: 0.65, and SCD: 0.60).

## Discussion

We investigated risk factors of adverse outcomes, including vascular dementia, sepsis, hospitalization for HF and MI, SCD, and all-cause death, in patients with new-onset AF using nationwide linked UK electronic health records. Our main findings are as follows: (i) the prediction models for adverse outcomes demonstrated fair to moderate predictive performance; (ii) some of the identified risk factors, such as smoking, and excessive alcohol consumption, are modifiable, and targeting them may help prevent major adverse outcomes. Hence, identifying individuals with AF at higher risk for cardiovascular and non-cardiovascular outcomes has the potential to enable earlier diagnosis, targeted prevention strategies, more personalized management, and better prognosis.

Multimorbidity is a common issue in the AF population, with patients frequently having multiple comorbidities.^17^ AF associates with a higher risk for adverse outcomes, such as stroke, vascular dementia, respiratory infections, HF, and SCD,^17,23,24^ suggesting that the prognostic trajectory of AF patients on a cardiovascular and non-cardiovascular level is fundamentally different from that of the general elderly population. Furthermore, this tends to be a frail and polymedicated population, requiring a more holistic and integrated management approach to improve the outcome.^25,26^ Despite the high incidence of various adverse outcomes in AF population, no integrated and holistic risk stratification schemes for conditions beyond stroke are currently available or implemented in routine AF care.

Our model identified several factors associated with an increased risk of several adverse outcomes, including older age, social deprivation, smoking, excessive alcohol intake, HF, stroke, venous thromboembolism, peripheral arterial disease, diabetes, chronic kidney disease, anaemia, and sepsis. Women showed lower risks of multiple adverse outcomes. These findings are consistent with previous studies reporting lower risks of MI, sepsis, and SCD in women.^27–29^ White ethnicity was associated with lower risks of HF and MI hospitalization, aligning with previous findings from a prospective UK Biobank cohort study showing that South Asian and Black participants had higher CVD risks compared with White participants.^30^ Obesity was associated with lower all-cause mortality, consistent with the so-called ‘obesity paradox’. One systematic review supports this phenomenon, whereby overweight and obese individuals with certain diseases may experience better outcomes than those with normal weight or underweight.^31^

Nevertheless, there are also discrepancies with previous reports. For instance, hypertension was associated with lower risk of all-cause death. Prior studies have demonstrated that hypertension increases the risk of all-cause mortality.^32^ However, a prospective US cohort study showed that treated and controlled hypertension was not associated with increased mortality.^33^ Treatment status was not captured in our analysis, which may partly explain this discrepancy. Finally, previous MI was associated with lower risk of sepsis. Whereas prior studies have shown that myocardial injury frequently occurs in patients with sepsis and is independently associated with early mortality and post-discharge cardiovascular morbidity,^34^ our findings may potentially be explained by survivor bias as frailer patients more likely to develop sepsis are more likely to die during an MI, and MI survivors may be more resilient and, hence, less likely to develop sepsis

Previous publications have shown that dementia, infections, and SCD are common in the AF population and responsible for high healthcare hospitalization and mortality.^17,23^ While previous studies have reported individual prediction models for HF and stroke in AF patients that despite good predictive performance provide a fragmented view of the complex AF patients, we developed an integrated prediction model that predicts multiple outcomes simultaneously, providing a more holistic view, using a combination of clinical variables that are routinely collected in clinical practice.^35,36^ Although advanced metrics from ECG, blood tests, coronary angiography, or CT and MRI imaging can enhance predictive accuracy, these tests are not commonly performed for all patients. By focusing on widely available data, our model ensures high clinical utility and broader applicability across diverse healthcare settings. To the best of our knowledge, no previous models have been developed to predict this wide range of outcomes based solely on clinical data. While the 2024 ESC guidelines for the management of AF emphasize that various adverse outcomes are associated with AF, they do not provide specific risk prediction tools for adverse outcomes beyond stroke.^4^ Our findings have important implications for clinical practice, as they may help identify high-risk patients who could benefit from further preventive strategies. The current lack of risk stratification schemes identifying individuals at low, intermediate, and high risk for diverse clinical outcomes following an AF diagnosis hinders the transition towards personalized medicine. Furthermore, a more granular understanding of risk profiles will facilitate cohort selection and the design of clinical trials to evaluate the efficacy of prognosis-modifying interventions in AF. Our study addresses this gap by developing models that predict these frequently associated outcomes using routine clinical variables. Our models may therefore help identify patients who require further assessment and preventive interventions, thereby contributing to improved outcomes. For MI, this could involve management of ischaemic risk factors such as smoking or anaemia and consideration of functional ischaemia testing when appropriate. With regard to HF, the models may support closer monitoring using echocardiography or biomarkers such as B-type natriuretic peptide, and facilitate timely initiation of evidence-based therapies (e.g. Angiotensin-converting enzyme inhibitors, Angiotensin-II receptor blockers and the other drug pillars of HF management). For vascular dementia, a meta-analysis has suggested that interventions targeting modifiable factors could reduce risk by up to 45%, highlighting the potential value of early intervention.^37^ International guidelines for management of sepsis and septic shock recommend screening, early therapeutic intervention, and initial resuscitation for patients with sepsis.^38^ Our risk prediction model may facilitate adherence to these recommendations by facilitating earlier identification and treatment of high-risk patients. Finally, for all-cause mortality, early identification of unrecognized conditions such as anaemia or cancer could enable appropriate interventions and improve prognosis. Our primary goal in this study was to establish and internally validate a robust predictive model. As a next step, we plan to perform external validation using independent nationwide datasets from America, Asia, and Europe to confirm the model’s generalizability and clinical utility.

An European Heart Rhythm Association (EHRA) perspective on digital data revolution and an international consortium highlight the significant potential of artificial intelligence (AI) in AF management.^39,40^ The ARISTOTELES project aims to develop AI-driven solutions for improving the clinical management of AF and plans to conduct a randomized controlled trial to assess the effectiveness of AI-informed patient management.^40^ While AI models show promise, the ‘black box’ nature of these algorithms remains a challenge. Integrating advanced AI techniques with interpretable prediction models, such as the one developed in this study, may provide a more effective and transparent framework for clinical decision-making.

### Limitations

A few limitations in this study warrant emphasis. First, using electronic health records may lead to unmeasured confounding because some variables related to adverse events could be missing. For example, information on medications or treatment history was not available, which may have influenced the observed associations. Second, we imputed missing values for variables such as smoking and ethnicity, under the assumption of missing at random. However, patients with missing data were generally older and had lower recorded prevalence of comorbidities. Complete-case analysis performed as sensitivity analyses showed similar predictive performance, supporting the robustness of our findings. Third, our study assessed associations rather than causality. As for every observational study, the possibility of reverse causation should be considered, although the temporal ordering between exposure and outcome was considered using time-to-event analyses. For example, patients with advanced comorbid conditions may present with lower blood pressure and a higher risk of death, which could explain the paradoxical association observed between hypertension and lower all-cause mortality. Fourth, the discrimination of some models was modest. In particular, risk prediction models for MI hospitalization, sepsis, and SCD showed relatively low c-index values, which could suggest limited utility as standalone tools. However, such c-index values are comparable to the ones observed for the CHA_2_DS_2_VASc score. As these models rely on routinely collected clinical variables, incorporating additional information, such as biomarkers (e.g. high sensitivity troponin for MI or procalcitonin for sepsis), may improve their predictive performance. Finaly, the generalizability of our results to current clinical practice remains uncertain since this study utilized data up to 2016 and the management of cardiovascular conditions has evolved significantly over the past decade with wider availability of treatment with direct OACs, catheter ablation, and novel drugs with prognostic impact on HF. Although the impact of these treatment options on specific outcomes, such as sepsis, MI, SCD, and vascular dementia, remains uncertain, the clinical variables used in our models remain highly relevant to the contemporary AF population. Nonetheless, future external validation studies involving modern cohorts are necessary to validate this prediction model before clinical implementation can be considered.

## Conclusion

In patients with AF, we identified several risk factors for adverse outcomes, including MI hospitalization, HF hospitalization, vascular dementia, sepsis, SCD, and all-cause mortality. Risk factors like older age, social deprivation, smoking, and excessive alcohol intake were associated with multiple of the assessed adverse outcomes. Our risk of the cardiovascular and non-cardiovascular outcome prediction models showed fair to moderate discrimination both in the derivation and validation cohort.

## Supplementary Material

oeag103_Supplementary_Data

## Data Availability

Data for this study were provided by United Kingdom’s Medicines and Healthcare Products Regulatory Agency following approval by the Independent Scientific Advisory Committee [17_205] and can be made available to other researchers following application via the CPRD website (https://www.cprd.com/).
